# A Relative Authorship Index: A New Metric for Evaluating Individual Contribution in Scientific Research

**DOI:** 10.1111/ejn.70464

**Published:** 2026-03-18

**Authors:** Francesco Siano, Mariella Segreti, Pierpaolo Pani, Emiliano Brunamonti, Aldo Genovesio

**Affiliations:** ^1^ Department of Physiology and Pharmacology Sapienza University Rome Italy; ^2^ Behavioral Neuroscience PhD Program Sapienza University Rome Italy; ^3^ Department of Pharmaceutical Sciences University of Piemonte Orientale Novara Italy

**Keywords:** authorship contribution, authorship index, bibliometrics, pybliometrics, research metrics, VQR

## Abstract

This study presents the relative authorship index (RAI), a novel metric designed to address the limitations of traditional bibliometric indicators, such as publication counts, citation numbers, and the *h*‐index, by correcting for authorship inflation. Conventional metrics can overestimate productivity by failing to account for the number of co‐authors or the possibility of inflated authorship. To detect such inflation, this index evaluates the number of co‐authors on a paper relative to the number of authors in the references cited within the same paper, which are assumed to reflect the researcher's specific field of study. By using this field‐specific baseline, the index identifies whether a publication involves an unusually high number of co‐authors compared to field standards, thus flagging potential authorship inflation. Applied to neuroscience articles authored by researchers affiliated with Italian universities, the index revealed significant regional and university differences, with higher values in southern regions and private universities. A case study of a single department also revealed high variability among individual researchers, indicating that the index can capture consistent patterns in authorship practices. In addition, we propose an *authorship correction formula* to adjust bibliometric indicators. The formula introduces parameters that penalize authorship inflation based on the RAI and also penalize large co‐author counts, with the latter scaled according to whether the researcher occupies key authorship positions (first, last, and/or corresponding author).

## Graphical abstract

This study introduces the relative authorship index (RAI) to assess individual contributions by correcting for authorship inflation. Analysis of the Italian academic landscape reveals that RAI is significantly lower in northern universities compared to central and southern institutions and lower in public versus private universities. Furthermore, a temporal analysis (sampled every 3 years) shows a steady upward trend in RAI over time, despite a slight decline around 2010, reflecting shifting collaborative patterns in scientific research.

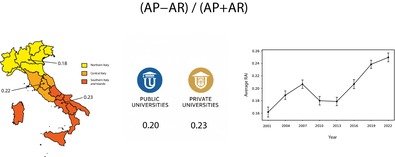

**Abbreviations**
ANVUR: Italian National Agency for the Evaluation of Universities and Research Institutes
DOI: digital object identifier
PhD: Doctor of Philosophy
RAI: relative authorship index
SEM: standard error of the mean
VQR: Valutazione della Qualità della Ricerca
WoS: Web of Science

## 1 Introduction

In recent decades, the number of co‐authors per paper has risen across many fields, a phenomenon often termed “authorship inflation” (Tilak et al. 2015; Lin and Lu 2023). For example, in biomedical research, the mean number of authors per publication increased from 3.99 to over 6 (+57%) between 2000 and 2020 (Jakab et al. 2024). Such growth in team size likely reflects, at least in part, the rise of large‐scale collaborations and multicenter studies (Chau et al. 2024); indeed, large collaborations are sometimes necessary to address the increasing complexity of scientific areas' experimental approaches and techniques (Abt 2007). For instance, contemporary neuroscience research frequently relies on complex technologies, computational or animal models of cognitive processes, global brain mapping initiatives, and advanced neuroimaging techniques (Segreti et al. 2025). Yet, this trend also raises concerns that traditional bibliometric indicators may over‐credit individuals in large collaborations, failing to distinguish each author's contribution.

In fact, conventional metrics like total publication counts, citation counts, and Hirsch's *h*‐index (Hirsch 2005) do not account for the number of co‐authors on each publication (Jain and Chandra 2025). Every co‐author of a paper receives full credit in these measures, as if they had authored the work independently (Perneger 2023). The *h*‐index, in particular, credits all co‐authors equally for each citation, creating no incentive to limit authorship to substantial contributors only (Perneger 2023). This means a researcher can boost their metrics by being listed on many papers, even with minimal input, because each paper's impact (citations) is fully counted for all authors.

Research confirms that inflated co‐authorship can distort performance indicators. In a cross‐sectional study based on 100 randomly selected papers from the MEDLINE database, Masic and Jankovic (2021) analyzed publications with more than 30 authors. They found that although such papers represented only around 10% of the sample, they accounted for over 40% of the total citations and contributed approximately 40% of the *h*‐index scores attributed to the researchers involved. The problem is that such metric inflation may not reflect true individual contributions or research quality (Masic and Jankovic 2021). Mora and Pilia (2024) calculated the number of publications and co‐authors of the Stanford top 2% researchers. Specifically, they reported publication rates of up to 212 papers and 5792 new co‐authors per year compared to a maximum of 28.3 publications and 173 new co‐authors per year among Nobel Laureates. Such high numbers were interpreted as suggestive of questionable publication practices (Mora and Pilia 2024).

Prior efforts to adjust for multi‐authorship have typically used fractional credit schemes or weighted author positions. For instance, Egghe's “Hm‐index” gives each paper fractional credit (1/*N* for *N* co‐authors) in an author's *h*‐index, so that adding more co‐authors yields diminishing returns (Egghe 2008; Schreiber 2008; Perneger 2023). Similarly, Onjia (2025) proposed a correction formula based on the number of authors and the position of authors. More recent approaches, such as the *h*‐leadership index proposed by Jain and Chandra (2025), refine this logic by assigning citation credit based on authorship position. Specifically, their index uses a modified complementary Gaussian weighting function, giving the highest weights to the first and last authors and progressively lower weights to middle authors. This allows the metric to capture researchers' roles in collaborative work and discourages reliance on middle authorship for inflating metrics. The result is a more equitable measure that acknowledges positional contributions without penalizing collaboration per se. Eriksson et al. (2018) proposed a mixed qualitative and quantitative approach, suggesting that researchers applying for positions be asked to explain a random selection of their co‐authored papers. This would complement the use of bibliometric indicators by dividing publications and citations by the number of co‐authors.

Publishing with a high number of authors can increase scientific output and provide a competitive advantage. The importance of considering the number of co‐authors when ranking researchers has been highlighted by ScholarGPS, which, unlike Clarivate's Highly Cited Researchers list, allows for author count weighting in its ranking methodology. Notably, publications with more than 20 authors are excluded, as it is stated that “in such cases it is often too difficult to ascertain individual contributions to the publication.” An Italian analysis of researchers across different disciplines, based on ScholarGPS data, was recently conducted by Ciriminna et al. (2025).

A large number of co‐authors may reflect the collaborative nature of a specific field or indicate authorship inflation, and ScholarGPS has the limitation of not addressing this issue. To distinguish between these two possibilities, it is necessary to evaluate the number of authors within the context of the specific field of study.

Our work introduces the relative authorship index (RAI), a new index to address this issue. It captures relative authorship inflation by contextualizing a paper's author count against a normative baseline drawn from its field or literature. In practice, the RAI considers how many co‐authors a given article has in comparison to the typical number of co‐authors in that article's cited references. This approach allows the RAI to identify cases where authorship appears unusually inflated compared to standard practices within the relevant field. A substantially higher number of authors than those listed in the cited literature may suggest that each contributor's share of the work is proportionally smaller, potentially indicating authorship inflation.

We calculated the RAI using neuroscience papers authored by Italian researchers as first or last author according to their listed affiliation to determine its distribution and average in Italy, which can then be used to assess individual researchers' RAIs. We also compared the number of authors and the RAIs in Italy across northern, central, and south and islands regions, as well as between public and private universities and, within private universities, between non‐telematic and telematic institutions.

We also investigated whether a high RAI reflects merely an occasional increase in the number of co‐authors or indicates a more consistent publication strategy leading to authorship inflation using a single university department as a case study. Our analysis revealed a highly heterogeneous distribution of personal RAIs, supporting the hypothesis that some researchers publish consistently with a disproportionate number of co‐authors beyond what would be expected based on the collaborative norms of their field. Therefore, we propose a corrective formula to apply to bibliometric indicators.

## 2 Methods

The article selection process was conducted using the Web of Science (WoS) platform by selecting papers published in journals dedicated to the field of neuroscience (a total of 306 journals). We retrieved all Italian articles published between 2001 and 2023, resulting in 25,662 articles in total. Specifically, an article was considered Italian if at least one Italian university appeared as either the first or the last listed affiliation. If only the first author was affiliated with an Italian university, we considered the first author for the analysis. If only the last author was affiliated, we considered the last author. If both were affiliated, we prioritized the last author. To identify such universities, we developed a mapping dictionary that cross‐referenced the English‐language names of Italian universities with their corresponding official Italian names. For instance, entries such as “University of Naples Federico II” were matched to their Italian counterparts: “Università degli Studi di Napoli Federico II.” This step was necessary because, whereas affiliations are most commonly reported in English in WoS, we observed that, in some cases, universities appeared in their original Italian form. Additionally, we manually checked how each university name was reported in WoS to avoid data loss. Taking “Università degli Studi di Napoli Federico II” as an example, we observed that, when listed in Italian, names were often reported without diacritical marks (e.g., without accents). As a result, we removed accents in the mapping to ensure that all affiliation variants were correctly recognized and included. Information about Italian universities was retrieved from the official website of the Ministero dell’Università e della Ricerca (n.d.).

In some publications, particularly those involving consortia or large‐scale collaborations, the number of listed authors exceeded 100 or even more. This was typically due to the inclusion of entire research labs or study groups as formal authors, where all individual members were counted (e.g., Gialluisi et al. 2021). To avoid distortions in author‐based metrics, we excluded all articles with more than 40 authors, following the methodology used in previous articles (Paul et al. 2024; Segreti et al. 2025). Furthermore, to avoid other biases from non‐standard publication types, we excluded reviews, meeting abstracts, and any document not classified as an original research article.

We then developed an index comparing the number of authors in a focal article to the average number of authors in its cited references. To compute this metric, we used the *pybliometrics* Python library (Rose and Kitchin 2019; pybliometrics·PyPI) to retrieve article metadata from Scopus via digital object identifiers (DOIs). For each article, we employed the *AbstractRetrieval* class from the pybliometrics.scopus module (pybliometrics.scopus.AbstractRetrieval—pybliometrics documentation). This class queries the Scopus API and returns a set of bibliometric information for the article identified by its DOI. When the *view = 'FULL'* parameter is specified, the output includes not only general article metadata (e.g., title, publication year, authorship, and source journal) but also the complete list of cited references. Each cited reference is returned as a structured object containing multiple fields, one of which is *authors*. This field is a single string that lists the authors of the cited reference in the format “Surname, Initials; Surname, Initials; …,” with each author separated by a semicolon. In some cases, authors appeared multiple times if they were affiliated with more than one university, due to the one‐to‐one mapping with affiliation data in Scopus.

**FIGURE 1:**
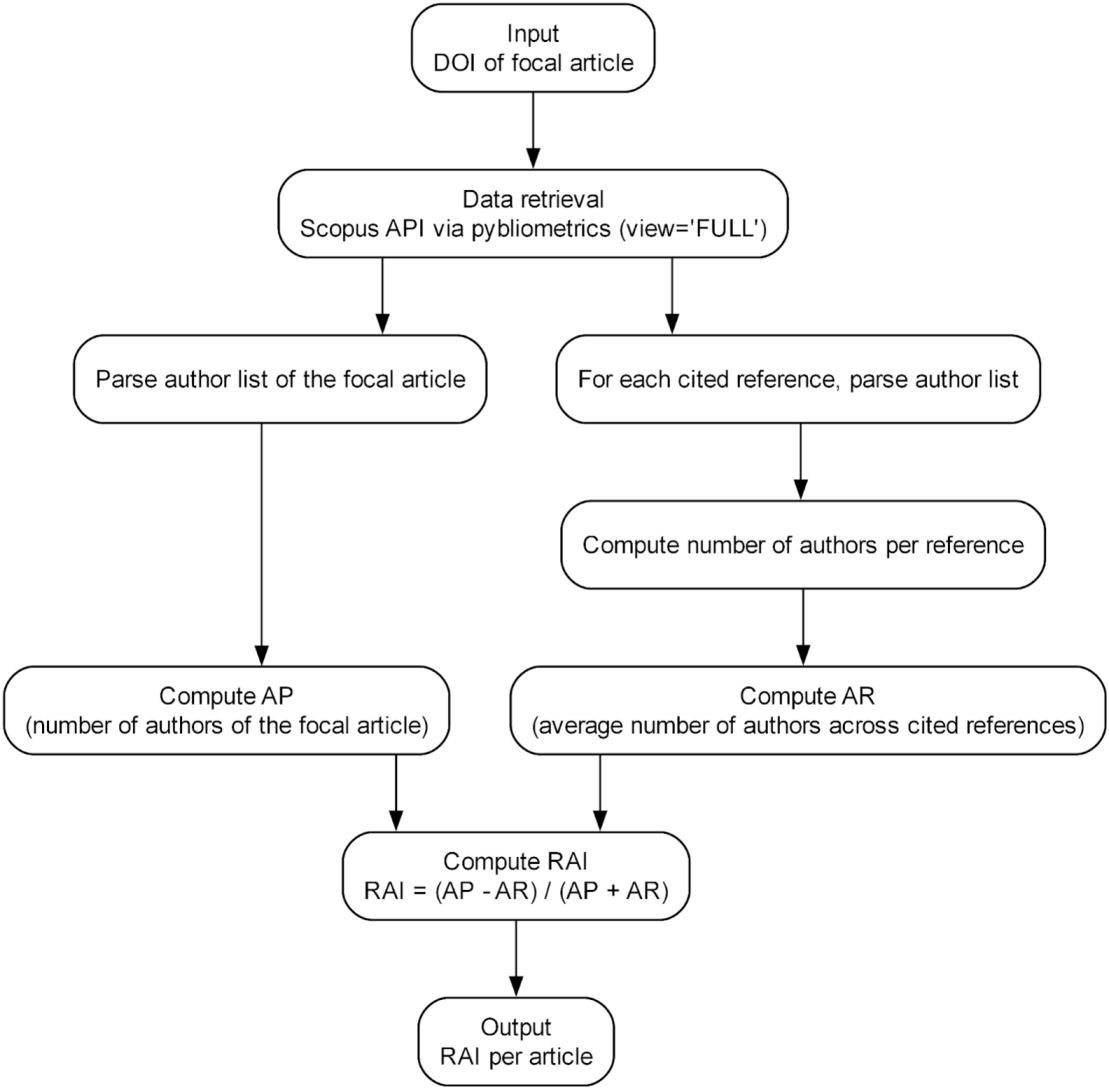
Diagram displaying each step needed to calculate an article's RAI starting from its DOI.

To determine the number of authors for each cited reference, we parsed the *authors* field by splitting the string at each semicolon and counting the resulting elements. We repeated this operation across all references in the article and calculated the average number of authors per reference. This allowed us to compute the RAI using the following formula:

$$\left(\mathrm{AP}-\mathrm{AR}\right)/\left(\mathrm{AP}+\mathrm{AR}\right)$$

where *AP* is the number of authors of the focal article and *AR* is the average number of authors among its cited references. The resulting value is a metric that reflects whether an article's authorship is inflated or conservative relative to the typical citation background. A positive index suggests higher‐than‐expected authorship (inflation), whereas a negative value suggests fewer authors compared to the reference baseline. The construction of the index is summarized in Figure 1.

After calculating the index for each article, we analyzed the distribution of index values by university type. In particular, we examined whether public versus private universities (including telematic universities) exhibited different patterns of authorship inflation as measured by our index. Additionally, we repeated the same analysis within the private universities field, comparing non‐telematic versus telematic universities. We also explored regional differences by grouping Italian universities according to their geographic location: north, center, and south and islands. This classification was performed manually by reviewing each university individually and assigning it to the appropriate region based on the official location of its main campus. Similarly, we manually identified the administrative status (public, private, and, within the private field, non‐telematic or telematic) of each university by consulting institutional websites, government databases, and ministerial classifications.

Finally, as part of a self‐evaluation effort, we focused on the Department of Physiology and Pharmacology “Vittorio Erspamer” at Sapienza University of Rome, to which several authors of this study are affiliated. We retrieved from the WoS all neuroscience articles published between 2001 and 2023 by department members who had produced at least 10 publications in the neuroscience field during this period. Our analysis included all *ricercatori* (assistant professors), *professori associati* (associate professors), and *professori ordinari* (full professors) affiliated with the department. Information about Department members was retrieved from the Sapienza University website (Università degli Studi di Roma “La Sapienza”, n.d.). Author names were identified from the department's official website and matched to WoS profiles using the Researcher search board option. Because we found multiple profiles with different affiliations for each author, we retrieved articles exclusively from those profiles that clearly listed our department as the main affiliation.

We set this criterion to avoid considering homonyms or articles not published as part of the overall department activity. We also excluded from the analysis all authors with fewer than 10 articles meeting the aforementioned criteria. This approach aims to avoid making evaluations based on too few publications. For each author, we calculated the RAI across their articles and further analyzed the average index over time. Finally, we ranked these authors based on their average RAIs, following the same procedure used in the previous analysis on Italian papers.

### 2.1 Analysis

Following the computation of the index, we examined differences in authorship patterns between public and private universities, as well as among universities located in northern, central, and southern Italy (including the islands). Within the context of private universities, we also analyzed the differences between non‐telematic and telematic universities. These analyses aimed to determine whether institutional or geographical factors were associated with systematic variations in authorship practices, as reflected by the index.

To extend the analysis to the individual level, we conducted a case study on the Department of Physiology and Pharmacology “Vittorio Erspamer” at Sapienza University of Rome. Here, we analyzed the department's authorship output. In this case study, the index evaluates researchers based on their overall publication output in neuroscience rather than individual publications, as in the previous analysis.

All data processing and statistical analyses were performed using the Python programming language (Python Software Foundation 2023). As shown in Table 1, we observed substantial interindividual differences within the department, indicating markedly different levels of authorship inflation.

**TABLE 1:** Ranking of the average RAI for each author of the Department of Physiology and Pharmacology “Vittorio Erspamer” at Sapienza University of Rome, with the uncertainty measures of the unbiased sample standard deviation.

Authors	Number of selected papers	Average RAI ± standard deviation (only neuroscience articles)
1	37	0.55 ± 0.14
2	51	0.50 ± 0.16
3	185	0.40 ± 0.17
4	18	0.38 ± 0.19
5	29	0.37 ± 0.16
6	26	0.35 ± 0.10
7	10	0.34 ± 0.20
8	13	0.32 ± 0.12
9	49	0.31 ± 0.16
10	170	0.29 ± 0.17
11	10	0.28 ± 0.12
12	14	0.24 ± 0.22
13	20	0.23 ± 0.17
14	25	0.23 ± 0.16
15	15	0.23 ± 0.15
16	14	0.21 ± 0.22
17	12	0.21 ± 0.12
18	21	0.21 ± 0.26
19	32	0.19 ± 0.17
20	13	0.18 ± 0.32

## 3 Results

**FIGURE 2:**
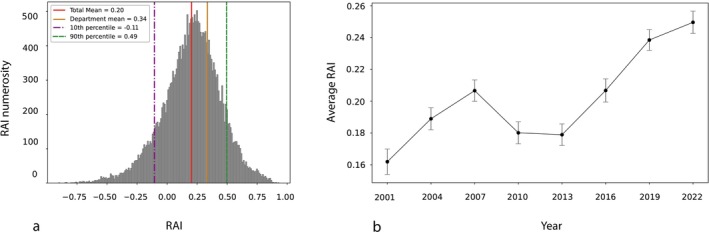
(a) Distribution of RAIs across Italy displaying the mean, the 10th and 90th percentiles, and the average RAI for the Department of Physiology and Pharmacology. (b) Temporal trend of the average RAI across Italy, with articles calculated every 3‐year intervals. Error bars represent the standard error of the mean (SEM), computed using the unbiased sample standard deviation.

**FIGURE 3:**
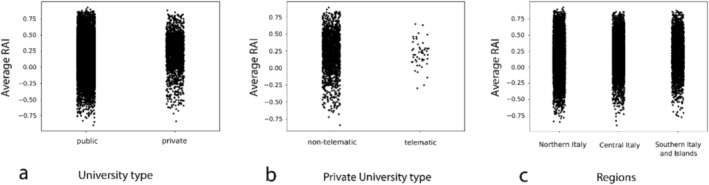
(a) Distribution of the RAIs by university type (public vs. private universities). (b) Distribution of the RAIs within private universities, distinguishing non‐telematic and telematic universities. (c) Distribution of the RAIs across the Italian regions.

Following the selection procedure described in Section 2, a total of 25,662 neuroscience articles with at least one Italian university listed as either the first or the last affiliation were identified. Throughout this analysis, we encountered a few articles for which *Pybliometrics* was unable to retrieve all the necessary metadata from the DOI. Consequently, it was not possible to calculate the RAI for these articles. Thus, metadata was successfully retrieved for 25,512 articles, and the RAI was calculated for each of them. The average RAI across the full dataset was 0.20, indicating a general trend toward slightly higher author counts compared to the average number of authors in the cited references (Figure 2a). To examine temporal changes in authorship practices, we calculated the average index every 3 years. As shown in Figure 2b, a clear upward trend in the average RAI was observed over time, with the exception of a modest decline around 2010. The analysis of authorship according to university type showed that articles from public universities had a lower average number of authors compared to those from private universities. Specifically, public universities reported a mean of 7.24 authors per article, whereas private universities showed a higher mean of 8.18. This difference was statistically significant, as confirmed by a Mann–Whitney *U* test (*U* = 20,946,503.50, *p* < 0.001). A similar pattern was observed for the RAI itself, with public universities showing a lower average (average RAI = 0.20 ± 0.0016) than private universities (average RAI = 0.23 ± 0.0059) (Figure 3a). The difference in RAI values was also significant (*U* = 22,160,658.50, *p* < 0.001), suggesting a greater degree of authorship inflation in private academic settings.

A focused comparison between non‐telematic and telematic universities (Figure 3b) revealed that, whereas non‐telematic universities had slightly higher mean values on both the number of authors per article and the RAI (8.22 authors, average RAI = 0.23 ± 0.0060) compared to telematic universities (6.87 authors, average RAI = 0.21 ± 0.026), only the number of authors reached statistical significance (*U* = 64,412.5, *p* = 0.0068).

Regional comparisons revealed additional variation. Articles affiliated with universities in northern Italy had the lowest mean number of authors (7.00), followed by those from central (7.45) and southern Italy, including the islands (7.89). These differences were statistically significant, according to a Kruskal–Wallis test (*H* = 351.28, *p* < 0.001). Post hoc comparisons with Bonferroni correction confirmed that all pairwise differences between regions were significant: north versus center (*p* < 0.001), north versus south and islands (*p* < 0.001), and center versus south and islands (*p* < 0.001).

When considering the RAI, the trend was similar (Figure 3c). Universities in the north showed a lower average RAI (0.18 ± 0.0022) compared to those in the center (0.22 ± 0.0027) and south (0.23 ± 0.0032), again showing a significant overall effect (Kruskal–Wallis *H* = 190.01, *p* < 0.001). Post hoc tests with Bonferroni correction confirmed significant differences for all comparisons: north versus center (*p* < 0.001), north versus south and islands (*p* < 0.001), and center versus south and islands (*p* < 0.001).

### 3.1 Department of Physiology and Pharmacology at Sapienza University of Rome as Case Study

We performed a department‐level analysis on the Department of Physiology and Pharmacology “Vittorio Erspamer” at Sapienza University of Rome, considering the articles published between 2001 and 2023. The analysis included only the departmental researchers who had authored at least 10 papers during this period in neuroscience. The overall departmental RAI average was 0.34 (Figure 2a), a value higher than the national average, but still within the 90th percentile, with individual values ranging from 0.18 to 0.55, as shown in Table 1.

### 3.2 Metrics Correction

We propose using the RAI to adjust researchers' bibliometric indicators, such as the number of publications, the *h*‐index, and citation counts, in proportion to their level of authorship inflation. For publication counts, the total number of publications produced by a researcher over their career can be corrected using the following *authorship inflation corrective formula*:

$$\text{Adjusted Publications}=\text{Original Publications}\times e^{-\alpha \left(\mathrm{RAI}-\mathrm{RAI}\_\text{field}\right)}$$

where *Original Publications* is the total number of publications, RAI is the researcher's average RAI, RAI_field is the average RAI within the relevant field (e.g., neuroscience), and *α* is a correction factor. For example, using *α* = 4, a researcher in neuroscience with 30 publications and an RAI of 0.403 (compared to an RAI_field of 0.203) has an adjusted publication count of 13.48. This implies that a researcher with an RAI equal to the field average would need at least 14 publications to outrank this researcher. With *α* = 10, the same researcher would have an adjusted publication count of 4.06, and a researcher with only 5 publications and an RAI equal to the field average would already outrank the researcher with 30 publications. Therefore, with *α* = 10, the correction might be too strong.

An additional correction can further account for the average number of authors per paper, independent of authorship inflation, by including an exponential penalty term in addition to the RAI correction term. In this case, the *authorship correction formula* becomes:

$$\begin{array}{c} \text{Adjusted Publications}=\text{Original Publications}\times e^{-\alpha \left(\mathrm{RAI}-\mathrm{RAI}\_\text{field}\right)}\\ \;\times e^{-\beta \cdot \left[\left(1-w\right)\cdot \left(N\_\text{authors}-1\right)\right]} \end{array}$$

Here, *N*_authors is the average number of authors per publication, *β* is the authorship penalty coefficient, and *w* is the proportion of papers where the researcher is first, last, or corresponding author (or shares these roles). If *w* equals 1, the researcher is always the first, last, or corresponding author; 0 if the researcher never holds these roles; and intermediate values between 0 and 1 based on the proportion of such authorship roles.

Using *α* = 4, *β* = 0.04, and *w* = 0, the first researcher with 30 publications, an RAI of 0.403, and *N*_authors of 20 would have an adjusted publication count of 6.30.

For comparison, the second researcher with 11 publications, *w* = 0, an RAI of 0.203, and *N*_authors of 6 would have an adjusted publication count of 9.01, which is higher than the previous case. In this case, the second researcher would have a higher adjusted publication number, even with fewer publications, because the first researcher has a higher RAI than the field average. Combined with the very high average number of authors, this results in a severe double penalty, considerably lowering the adjusted publication number.

With the same *α* = 4 and a smaller *β* = 0.01, the first researcher would outrank the second, with an adjusted publication count of 11.15 versus 10.46 for the second researcher, because the penalty associated with the number of co‐authors is substantially reduced. Interestingly, if *w* = 0.8, keeping *α* = 4 and *β* = 0.04 for both researchers, the first researcher would outrank the second with an adjusted publication count of 11.58 versus 10.57, reversing the previous ranking obtained with *w* = 0.

The application of the *authorship correction formula* provides an estimate of individual scientific contribution by accounting for authorship inflation, the average number of co‐authors, and the author's role in each publication.

## 4 Discussion

The index introduced in this study provides a new metric for evaluating individual scientific contributions in academic recruitment and evaluation processes. Academic committees often rely on bibliometric indicators (number of publications, citations, *h*‐index, etc.) as part of their assessment of candidates. Yet, as Perneger (2023) has argued, these metrics systematically attribute the same credit to all co‐authors, regardless of their role or position in the author list. This equal treatment makes conventional indicators vulnerable to distortion, whether through inflated co‐authorship lists or participation in large collaborations where individual contributions are minimal.

Our index addresses this limitation by capturing relative authorship inflation. Specifically, it contextualizes the number of co‐authors of an article against the normative background provided by its cited literature and can be used to correct the bibliometric indicators.

We have used the cited articles as the reference to calculate the RAI, which provides a fixed baseline for evaluating an article. However, as an alternative or in addition, we could have used the number of authors in the citing papers or the co‐citation network, as defined by Hutchins et al. (2016). In their work, the co‐citation network is composed of all articles that are cited alongside a given reference article by subsequent papers citing the evaluated paper. This network reflects the article's field, and they used the co‐citation network to normalize the citation counts of the evaluated article (Hutchins et al. 2016). Such a network is advantageous when it comes to evaluating a paper's influence over time in a dynamic and field‐sensitive way, but it is unnecessary for the calculation of the RAI.

Using the RAI might enhance the fairness of selection criteria in hiring competitions. For instance, if Researcher A and Researcher B both wrote papers with 20 co‐authors in neuroscience, but Researcher A works in a subfield where small‐team publications are the norm (resulting in a high RAI, indicating inflation), whereas Researcher B operates in a subfield where large teams are standard (leading to a low RAI), then a simple normalization by the number of authors would fail to capture the authorship inflation of Researcher A, whereas the RAI would effectively do so.

In academic hiring and research evaluations, authorship inflation has often been the “elephant in the room,” a well‐known issue but never or rarely addressed. In this context, identifying the extent of a candidate's co‐authorship inflation can help prevent the overestimation of contributions from individuals who may have played only a minor role in large‐scale collaborative projects.

The value of this adjustment becomes even clearer when considering a common scenario in academic evaluation: Without such contextual information, a junior scientist who happened to be 1 of 50 authors on a few highly cited papers could unfairly outrank a solo researcher with slightly fewer citations. Indeed, Masic and Jankovic (2021) analyzed publications with more than 30 authors, and they found that although such papers represented only around 10% of the sample, they accounted for over 40% of the total citations and contributed approximately 40% of the *h*‐index scores attributed to the researchers involved.

The RAI could also be applied in evaluating the scientific output of the university departments in Italy for national research assessments. In Italy's *Valutazione della Qualità della Ricerca* (VQR), a national evaluation exercise managed by ANVUR (the Italian National Agency for the Evaluation of Universities and Research Institutes), each researcher is required to submit a given number of their publications for evaluation.

The RAI would provide a quantitative framework that evaluates the number of authors relative to field‐specific authorship for the article presented.

In Italy, departments have been competing to be among the selected 180 Departments of Excellence, which ensures increased funding. On a more aggregate level, the RAI can offer insights into publication practices at the group, departmental, or institutional level. A higher average RAI for a department would indicate that, on the whole, its publications involve more authors than is typical in their respective fields. This might reflect a culture of extensive collaboration (e.g., participation in multicenter trials) that exceeds what is common in the field or potentially a propensity for honorary co‐authorship. In either case, such information flags where publication practices deviate from the norm and warrants a closer look. Mora and Pilia (2024) proposed normalizing author metrics through a multiplicative adjustment aimed at penalizing the advantages gained from exceptionally high publication and new co‐author rates. However, their method is not effective in detecting authorship inflation and does not correct the number of authors on a smaller scale. In contrast, our index is scale independent and can detect authorship inflation at both low and high levels of publications and co‐authorship.

We also compared the number of authors and the RAI across northern, central, and southern (including the islands) Italy and found that both metrics increased from north to south. Additionally, we observed a significant difference in the number of authors between public and private universities, with private universities showing the highest number of authors.

We believe that a RAI, or any analogous index, should be considered in any fair research evaluation process. However, when researchers have similar RAIs, differences in the actual number of co‐authors may still provide meaningful information. In some cases, papers with more authors may genuinely require larger teams with diverse expertise. Even so, the total number of co‐authors should be taken into account when evaluating a researcher's output, particularly if the time and effort invested by each contributor tend to decrease as team size grows. If individual contributions diminish with increasing co‐authorship, then research fields traditionally relying on smaller teams face a potential disadvantage. Their overall output might appear lower, even if the individual effort is comparable or greater. Over time, this could lead to their underrepresentation or even extinction within competitive academic systems.

By providing the script to calculate the RAI and the *authorship correction formula* for both authorship inflation and the number of authors, we hope they can be used to evaluate the scientific output of both individuals and organizations, especially in comparative assessments. We have also proposed weighting each publication of a researcher, considering the contribution of the authors categorically as important or less important based on their position on the author list and on their corresponding author role. However, the *authorship correction formula* could also be adapted using non‐categorical criteria for defining the contribution of authors and calculating the *w* coefficient. The credit among authors does not necessarily have to be categorical but can instead vary, for example, between 0 and 1, as proposed by Konar (2021) for weighting citations. In Konar's approach, the corresponding author should be responsible for assigning a fraction of the credit to each contributor that should not exceed 1 in total. The problem with this approach is that its implementation must be on a large scale to be usable.

Similarly, Zerem (2017) proposed the Author Contribution Score, a metric for quantifying individual authorship as a fraction of the total credit. In this system, the first author receives the full allocation of points, whereas the corresponding author, if different from the first author, receives half of the total points. The remaining half is distributed equally among all other co‐authors. When the first author also serves as the corresponding author, the full point allocation for that role is shared equally among the remaining authors as specified. Notably, their index does not assign any particular importance to the last author in the evaluation. Our correction formula is flexible and could be adjusted to assign different penalties to the inflation of authorship and to the number of authors per se, weighted by the author role. One possibility for setting the parameters of the authorship correction formula is to consult the scientific community, and we expect that the optimal parameters would vary across fields.

Future studies should calculate the average RAI for each of the different research sectors, which in Italy are organized into *settori disciplinari*, to test whether they differ and thus support fair and accurate assessments of individual researchers. More importantly, the average RAI should be calculated separately for each country to provide a country‐based reference, as it is expected to vary with the number of authors (Segreti et al. 2025). Similarly, for grant evaluations, the average RAI should be calculated based on the relevant ERC sectors. In Italy, neuroscience is not classified as a distinct sector, so our index may not have immediate direct application; however, it can serve to promote the adoption of this form of evaluation across all types of research assessments. It is important to calculate the average national RAI because the baseline index is not expected to be zero. This is unsurprising, as recent articles also cite older studies characterized by a smaller number of authors (Segreti et al. 2025) and fields that primarily cite recent work might therefore display a smaller RAI.

More in‐depth studies should examine the evolution of academic careers to assess whether a high RAI influences the speed of career advancement, such as the time taken to reach an assistant professor position after completing a PhD, or the duration between academic ranks leading up to full professorship.

In conclusion, we believe that whenever a comparative evaluation is needed, both at the individual level and at the group level, differences in RAI, or a similar index, should be taken into account, especially in countries with a high number of authors, such as Italy (Paul et al. 2024; Segreti et al. 2025). The broad adoption of the RAI would ultimately help discourage the inclusion of undeserving authors.

### 4.1 Limitations

Identifying the appropriate filtering criteria in Scopus to select articles specific to each sector presented some challenges. The overlap of research topics across multiple fields can lead to inconsistencies or misclassification, thereby affecting the reliability of the RAI when applied beyond the neuroscience domain.

Additionally, as described in Section 2, some technical limitations of *Pybliometrics* in retrieving metadata from the Scopus API may have led to an overestimation of the number of authors per reference. Furthermore, *Pybliometrics* is able to find only articles stored in the Scopus database; therefore, the passage from WoS to Scopus might lead to a loss of some articles. With regard to our department analysis, a further limitation is that, due to our strict filtering criteria of each author's articles from WoS, the actual total number of papers published by each author might be underestimated. Indeed, we needed to establish a standardized procedure to minimize the risk of including homonyms or papers not affiliated with our department in the analysis, which could otherwise distort the estimations.

## Author Contributions

**Francesco Siano:** conceptualization, data curation, formal analysis, methodology, software, validation, visualization, writing – original draft, writing – review and editing. **Mariella Segreti:** conceptualization, formal analysis, methodology, software, validation, visualization, writing – original draft, writing – review and editing. **Pierpaolo Pani:** conceptualization, data curation, formal analysis, methodology, supervision, validation, writing – original draft, writing – review and editing. **Emiliano Brunamonti:** conceptualization, data curation, formal analysis, methodology, project administration, software, supervision, validation, visualization, writing – original draft, writing – review and editing. **Aldo Genovesio:** conceptualization, data curation, formal analysis, methodology, project administration, software, supervision, validation, visualization, writing – original draft, writing – review and editing.

## Conflicts of Interest

The authors declare no conflicts of interest.

## Supporting information


**Data S1:** Supporting Information.

## Data Availability

The entire dataset used and all the Python scripts developed for the analysis are available in an open‐access repository (https://github.com/Arthemis98/Relative‐authorship‐index/tree/main).
